# The Analysis of bcl-2 in Association with p53 and Ki-67 in Triple Negative Breast Cancer and Other Molecular Subtypes in Ghana

**DOI:** 10.1155/2021/7054134

**Published:** 2021-06-03

**Authors:** Charity Ameh-Mensah, Babatunde Moses Duduyemi, Kweku Bedu-Addo, Elijah Atta Manu, Francis Opoku, Nicholas Titiloye

**Affiliations:** ^1^Department of Physiology, School of Medicine and Dentistry, Kwame Nkrumah University of Science and Technology, Kumasi, Ghana; ^2^Departments of Pathology, School of Medicine and Dentistry, Kwame Nkrumah University of Science and Technology, Kumasi, Ghana; ^3^Department of Anatomic Pathology, University of Sierra Leone Teaching Hospital Complex College of Medicine & Allied Health Sciences, Freetown, Sierra Leone

## Abstract

**Background:**

Little is known about the role of apoptosis in the tumorigenesis and prognosis of breast cancer in Ghana. Chemotherapeutic drug efficacy partially relates to apoptosis induction, rendering it a vital target in cancer therapy with unique biomarker opportunities that have not been exploited. Aberrations in this pathway are central to tumorigenesis, tumor progression, overall tumor growth, and regression during treatment therapies. Antiapoptotic bcl-2 (gene) and p53 are known to play roles in apoptosis while Ki-67 is a proliferative marker. The aim of our study is to determine the association of bcl-2 (protein) with p53 and Ki-67 in 203 consecutive breast cancer cases over a 10-year period.

**Method:**

A retrospective cross-sectional study on archival FFPE tissue blocks over a 9-year period with abstraction of clinicopathologic data. Two hundred and three consecutive and suitable FFPE blocks were selected for tissue microarray (TMA) construction, and IHC (bcl-2 (protein), Ki-67, p53, cyclin D, pan cytokeratins A and E, ER, PR, and HER2/neu) was done. Expressions of bcl-2 (protein), p53, and Ki-67 were related to histological grade, lymphovascular invasion, and molecular subtypes. SPSS version 23 was used to analyze results.

**Results:**

Most of our cases were in the fifth decade of life (31%); invasive carcinoma of no special type (NST) was predominant (87%); histological grade III (38%) was the highest; and Luminal A (19.8%), Luminal B (9.9%), HER2 (16%), and TNBC (54.3%) constituted the molecular classes. bcl-2 expression was found in 38% of the cases. Our cases also showed mutation in p53 (36.7%) and ki-67 expression (62.5%). bcl-2 (protein) and p53 significantly correlated with Luminal B and TNBC (*p* < 0.01). Ki-67 also correlated significantly with Luminal A and B and HER2 overexpression (*p* < 0.01). Premenopausal age (40–49) and histological grade inversely correlated with bcl-2 (protein) expression. p53 statistically correlated with Ki-67 (*p* < 0.05).

**Conclusion:**

Our results show high expression of bcl-2 (protein) suggesting an important role of apoptosis in Ghanaian breast cancer cases. bcl-2 (protein), p53, and Ki-67 expressions emerged interdependently from this research and can thus be manipulated in prediction and prognosis of breast cancers in our setting.

## 1. Introduction

Noticeable alterations in the breast occur related to proliferation, differentiation, and apoptosis through the cell cycle. A balance of these processes is crucial for homoeostasis and development with dysregulation likely to initiate tumorigenesis. Worldwide, cancer of the breast tissue is the most common invasive cancer affecting about 12% of women [1] and, occasionally, men. It affects older women but it is being seen in relatively young African females [2], probably due to late detection and poor diagnosis. Africans present high grades of breast cancers than Caucasians [3]. Breast cancer is the most common cancer in women and also the most common in the general population in Ghana. The estimated new cases were 4482 constituting 18.7% of total cancer in 2020 in both sexes (Globocan, 2020) [4]. The female gender, early age at first menstruation, obesity, lack of physical exercise, alcoholism, ionizing radiations, hormone replacement therapies in menopause, old age, having children late or not at all, and family history of breast cancer are risk factors. Diagnosis is confirmed by laboratory examinations of biopsy to ascertain the type of cancer, invasion, and metastasis as well as treatment options. Prognosis largely depends on these with respect to the person's age. Survival rates are better in the developed world [5].

Complex error correction and prevention measures innate to the phases of the cell cycle (Go, G1, S, G2, and M) safeguard the cell. Significant errors occur but damaged cells self-destruct by apoptosis. However, some mutations pass to progeny when the apoptotic pathway is defective leading to accumulation and tumorigenesis [6]. The mutations are related to environmental factors (90–95%) in genetically susceptible hosts [7], with 5–10% inherited from parents, including Breast Cancer Associated Genes (BRCA) 1 and 2 [8]. The apoptotic pathway of cell death is characterized by distinct morphological characteristics. Control and regulation are influenced by members of the family of bcl-2 (protein) [9]. Changes in regulation by the bcl-2 family occur partly under the influence of hormones [10]. Other intercellular and intracellular proteins actively involved in the biochemical processes of cell proliferation and apoptosis in multicellular organisms include the Ki-67, p53, cyclin D, and cytokeratins [11].

Histology and immunohistochemistry (IHC) have helped to group breast cancers based on biological behavior and interactions with known factors for prediction, prognosis, and appropriate clinical management [12]. These techniques have helped to detect substantive DNA damage and other cellular process defects that caused mutations bridging traditional, clinical, pathological, predictive, and prognostic biomarkers to improve healthcare [13].

This study sought to identify some apoptotic and regulatory proteins involved in breast cancers of women diagnosed in Komfo Anokye Teaching Hospital, Kumasi. For this purpose, immunohistochemistry analysis of ER, PR, HER2, p53, and Ki-67, as well as bcl-2 (protein) as an antiapoptotic protein, is considered. Proliferation enhancement and inhibition/induction of apoptosis by these proteins were implicated in tumorigenesis. Their frequencies, roles, and effects on each other in 203 persons with breast cancer were studied and recommendations given. Breast cancers of our subregion have been grouped in standard molecular classes to help prognosis.

The role of apoptosis in physiology cannot be overemphasized as it checks the activities of mitosis and cell proliferation in the regulation of cell populations. These biomarkers (Ki-67, bcl-2 (protein), and p53) have been studied in prostate, melanomas, and other cancers in the general population [14–16].

## 2. Methodology

This retrospective cross-sectional study was conducted on breast cancer reported, examined and diagnosed at Komfo Anokye Teaching Hospital (KATH), Kumasi, from January 2009 to December 2017. Our hospital is a referral center which serves the northern sector of Ghana and its neighbouring countries. Therefore, this study fairly covered a substantial population of the country and may represent the Ghanaian populace diagnosed with breast cancer.

Approval was obtained from the Committee on Human Research, Publications and Ethics, KNUST School of Medicine and Dentistry (CHRPE/AP/417/18), and the Research and Development Unit, Komfo Anokye Teaching Hospital (Reg. No. RD/CR18/203) on “Molecular Profiling of Breast Cancer in Kumasi.”

Demographics including age, gender, site of biopsy, histological diagnosis, histological grade of tumors, and lymphovascular invasion were abstracted from request forms, reports, and surgical daybook. Formalin fixed and paraffin embedded (FFPE) tissue blocks were collected from the archives. The tissue blocks collected were physically examined for the presence of viable tissues. Hematoxylin and eosin (H&E) stained slides were made and evaluated microscopically to confirm histological diagnoses of breast cancer. Tumors were classified using the WHO classification of breast cancers and graded by the Nottingham Grading System [17, 18]. Slides with representative sections of malignancies and their accompanying blocks were recruited for the study.

Each recruited breast tissue specimen conservatively sampled was given a new code for easy identification during the TMA recipient block construction. Representative blocks and slides were matched. Recipient paraffin blocks were made with a silicone mold supplied with a manual TMA machine (Micatu MicaArray Gen. 4). Two separate forty-eight 0.6 mm cores were excised from each with a pipette. Two 0.6 mm cores were then excised from every tumor (donor) block and released into adjacent recipient cores. Two different pipette nozzles (a small but long one and a wide but short one) were used. The small but long nozzle excised recipient cores while the wide but short one excised donor cores. The two adjacent donor cores on recipient blocks ensured that adequate tumor sections were selected, and results of all samples were confirmed. Samples of two hundred and three malignant breast cancer tissues were therefore finally reconstructed into 5 recipient tissue blocks using a manual tissue microarray (TMA) with an average of 40 tumor positive cases per block. These were heated briefly by an incandescent lamp to soften the paraffin, thereby allowing the tissue cores fit in better.

Fresh 4 *μ*m representative sections were cut from each block onto Superfrost Plus slides. Eight different slides were made from each recipient block and immunohistochemically stained according to the standard procedure. Recipient slides were conducted through citrate buffer at pH specific to type of primary antibody and microwave heated for 10 min. Sections were incubated for 5 min in 3% H_2_O_2_ and washed with water. Drops of mouse monoclonal primary antibodies were introduced and incubated at room temperature for 60 min. Immunoreactivity during incubation resulted in the expressions of the antigens under study (bcl-2, p53, Ki-67, ER, PR, HER2/neu). Drops of DPX Mountant were meticulously applied to stained slides to prevent trapped air bubbles. Microscopy was done after slides dried up (Table 1).

Tumor cells were deemed positive for bcl-2 (protein), mutant p53, ER, PR, and Ki-67 when there was unequivocal nuclear staining in at least 1% of the tumor section. Membrane staining was used for HER2/neu scores according to ASCO/CAP guidelines [17]. Ki-67 expression was categorized as low (<6%), moderate (6–10%), and high (>10%) [18]. In homogenously stained slides, 3 high power fields are selected randomly from sample edges for Ki-67 count. This was done in anticipation of possible biological heterogeneity across samples and actively invasive peripheries. Four molecular classes were derived based on slight modifications of methods described by Carey et al. [19]: triple negative (ER−, PR−, HER2−), Luminal A (ER+ and/or PR+, HER2−), Luminal B (ER+ and/or PR+, HER2+), and HER2 enriched subtype (HER2+, ER−, PR−). The IHC scoring was done by two pathologists independently according to ASCO/CAP guidelines [17].

### 2.1. Statistical Analysis

Data were given new codes, tabulated, and analyzed with SPSS version 23 for estimation of associations between variables. Chi-squared test (*χ*^2^-value) compared categorical variables. At 95% confidence interval, *p* values < 0.05 were statistically significant.

## 3. Results

Two hundred and three (203) malignant cases with tumor representative sections on preserved blocks were used for the study. The mean age of the cases was 49.34 years, and invasive carcinoma of no special type (NST) was the most recorded histological diagnosis (83.0%). More than half of the cases presented with histological grade III (55.7%), and 50.8% of 65 recorded cases were negative for lymphovascular invasion as shown in Table 2.

### 3.1. Immunohistochemistry (IHC)

**Figure 1:**
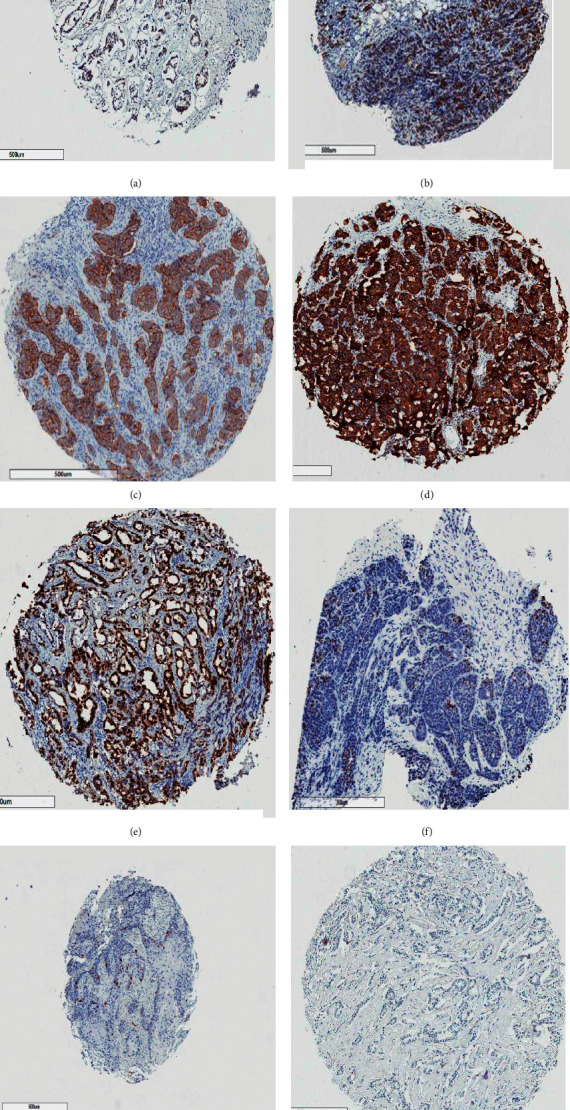
Photomicrographs of cases positive for ER (a), PR (b), HER2 (c), bcl-2 (d), p53 (e) Ki-67 high (f), Ki-67 medium (g), and Ki-67 low (h) immunohistochemistry. The golden brown appearances of tumor cells signify positive areas.

bcl-2 (protein), the primary protein of interest in this research, was positively stained in about 37.9% (77 out of 203) of the cases. Most cases failed to express the traditional and conventional hormone receptors for estrogen, progesterone, and human epidermal growth factor. The other markers studied had varying expressions. p53 expression was seen in 36.7% of cases and Ki-67 in 62.1% as presented in Table 3. The DCIS was excluded from IHC. The IHC photomicrograph of various antibodies is shown in Figure 1.

Cases were stratified into the four main molecular subclasses from ER, PR, HER2, and Ki-67 expressions. The triple negative (54%) was the most common phenotype and Luminal B was the least expressed as seen in Table 4.

### 3.2. Tests for Associations

The Chi-squared test established associations between bcl-2, p53, Ki-67, the molecular phenotypes, and the clinicopathological characteristics. A *p*-value of <0.05 was considered significant. bcl-2 statistically significantly correlated with the Luminal B and TNBC (*p*=0.015 and *p*=0.038, respectively). Histological grade at presentation, Luminal B, TNBC, and Ki-67 statistically significantly correlated with p53. Luminal A and B, HER2 positive, and p53 correlated significantly with Ki-67 as shown in Tables 5 and 6.

## 4. Discussion

Growth or regression of tumor during treatment depends on the rates of proliferation and apoptosis within the cells of the tissue [20] with most available therapies inducing apoptosis partly [21]. Therefore, this study seeks to categorize breast cancer with respect to apoptosis using IHC so as to understand the role of apoptosis in breast cancer in our environment. Literature has highlighted the effect of bcl-2 (protein), p53, and Ki-67 biomarkers on uncontrolled proliferative and apoptotic pathways in breast cancers [20, 21].

Our population is of African descent, and cases within the perimenopausal ages were more affected. The fifth decade recorded the most number of malignancies, similar to other studies in Ghana [22], Pakistan [23], and USA [24]. Most of our patients are premenopausal, and it has been postulated that this group have poor outcome due to dysregulated endocrine functions and hormone imbalances from hormone-based birth control methods [25]. Late detection and poor diagnosis are also reasons for high grades at presentation in our study. This is in agreement with the findings of other studies [2, 7].

The most common malignancies are the invasive carcinomas of NST (87.12%) and ductal carcinoma *in situ* (3.68%), similar to other studies from Africa [26, 27], Brazil [28], and England [29]. More than half of the cases were of grade 3 and very few were of grade 1, which agrees with a study by Stalk et al. [30] but contrasts with another by Eugenio et al. [28]. High grade tumors have poor prognosis [31, 32]. This suggests that a greater proportion of our patients may die from breast cancer and its related complications, supporting the low survival rates in developing countries [7].

In this study, about half of our cases showed lymphovascular involvement or invasion which primarily indicates poor prognosis [28, 33]. The number of affected lymph nodes, which we have no data on, may as well be of prognostic importance.

Breast cancer is a heterogeneous disease whose subtypes have specific biology and history. Identifying these subtypes is required to well manage this disease due to the challenge of predicting prognosis accurately and deciding precisely the best therapy to use. Positive lymph node counting and grade of tumor at presentation were heavily relied on. The Nottingham Prognostic Index (NPI) is validated, standard, and independent prognostic factor commonly used in breast cancer. Immunohistochemical studies on tissue microarrays have therefore become practical alternatives to improve breast cancer classification and prognosis [34]. However, IHC is not routinely done in developing countries like Ghana despite the invaluable prognostic and therapeutic information it provides because of difficulties in securing antibodies coupled with its high cost.

The expressions of the markers (bcl-2, p53, Ki-67, ER, PR, HER2, cyclin D, and pan cytokeratin) immunohistochemically studied are proportionally relevant in the cohort. Alterations in the expression patterns of bcl-2 family are expected in the malignancies since dysregulated apoptosis and reduced or inactive p53-ribosome signaling are known pathways in neoplasia. Antiapoptotic bcl-2 increase or positivity supports survival of damaged cells by blocking growth factors that ensure apoptosis (release of cytochrome c) or slowing proliferation so that cell numbers increase over long periods of time [35], resulting in tumor formation [36]. bcl-2 (gene) expression is positively influenced by estrogen (which stimulates proliferation of mammary cells whose increased rate of replication exposes both cells and DNA to mutations from genotoxic wastes accumulated [37]) but inversely related to TP53 gene mutation [38]. bcl-2 positivity has also been linked to differentiation, which largely depends on estrogen and low proliferation [35], to predict favorable outcome [39]. The bcl-2 (protein) was overexpressed in 37.9% of our cases, revealing a compromised apoptotic pathway as a relative cause of breast neoplasia in our environment. The low percentage recorded may be due to the relatively low sample size.

The TP53 gene, a commonly mutated gene in human cancers, regulates DNA repair through the cell cycle and apoptosis [40]. Overexpression of p53 has been correlated with poor outcome [38]. Any mutation of the TP53 gene (36.7% recorded in this study) has a potential to alter the apoptotic pathway and result in tumorigenesis [11]. Ki-67 marks how frequently tumor cells proliferate. They are found mostly in malignant and poorly differentiated cells [41]. The 62.5% recorded in this study shows the high rate of proliferation of breast cancer in our setting although the apoptotic pathway was not completely defective, accounting for the high number of cases with grades II and III at presentation. Most of the cases however expressed low Ki-67.

The triple negative breast cancer (TNBC) subtype (53.4%) was the most common phenotype from the study similar to most studies in our region with a range of 15.8–84% [3, 42]. TNBC has been found to be associated with the high grade, more advanced stages at diagnosis, and very few treatment options because they lack drug-targetable receptors which affect response rates and outcome [34]. They are the most sensitive molecular subclass to chemotherapy [43, 44]. There was a significant correlation between bcl-2 expression and TNBC in our study which may be a pointer to good survival outcome [45], contrary to many other studies which correlated low bcl-2 expression with poor prognosis in TNBC [46, 47], though survival outcome was not included in this study. However, TNBC had the highest proliferative index [48] due to its high Ki-67 percentage [49]. From these, we deduce that the high histologic grades at presentation and high Ki-67 percentage coupled with a positive significant antiapoptotic bcl-2 (protein) correlation account for the proportionally large aggressive phenotype observed in our cohort [50].

The Luminal B subtype could be aggressive because of the effects HER2 confers. The significant association between bcl-2 (protein) and Luminal B might lead to reduced susceptibility to anti-ER therapy by breast cancer cases in our region [37, 51].

The bcl-2 (protein) expression and its prognostic role differ according to molecular subtypes and is a good prognostic marker for mainly Luminal B and TNBC, which are known to be more clinically aggressive and expected to have poorer prognoses [52]. The Luminal B and TNBC molecular subclasses correlated statistically with p53, as well as the high histological grades at presentation. These portend poor prognoses such as carcinogenesis will progress because more cells will escape control, survive, and replicate [38]. Luminal A and B and HER2 positive molecular subclasses correlated statistically with Ki-67. These groups will benefit from endocrine and HER2-targeted therapies like tamoxifen, aromatase inhibitors, and trastuzumab to slow down proliferation [53]. Ki-67 also statistically correlated with p53 mutation, suggesting dysregulation of the cell cycle to override apoptosis and ensure overproliferation [54].

Low rates of programmed cell death (apoptosis) or high levels of antiapoptotic protein bcl-2 (protein) will result in poor prognosis though some studies have implicated frequent and higher rates of apoptosis in malignancies [55]. This suggests some measure of control in the regulation of both proliferation and apoptosis in tumorigenesis. Generally, there was a moderately high rate of apoptosis (antiapoptotic bcl-2 effect was 37.9%) from our study. The rate of apoptosis, which is often reduced in cancers, could also be due to decreased proliferation rates caused by bcl-2 (gene) on Ki-67 activities and has been related to poor survival [13, 39, 56].

The bcl-2 (protein) expression in this study can be linked with hormone receptor expression, low proliferation (62.1%), and TP53 gene mutation (36.7%) expressions suggesting better intrinsic prognosis [57]. This suggests that bcl-2 played an antiproliferative role despite being antiapoptotic [58], thereby suppressing tumorigenesis in association with other biomarkers.

## 5. Conclusion

The bcl-2 (gene) played both antiproliferative and antiapoptotic roles in tumorigenesis. Our results show high expression of bcl-2 suggesting an important role of apoptosis in Ghanaian breast cancer cases. bcl-2, p53, and Ki-67 expressions emerged interdependently from this research and can thus be manipulated in prediction and prognosis of breast cancers in our setting. The association between TNBC and bcl-2 (protein) highlights possible therapeutic targeting of bcl-2 (gene) for TNBCs.

## Figures and Tables

**Table 1:** Details of antibodies used.

Antibody	Clone	Pretreatment	Dilution	Control	Company	Address
bcl-2	bcl-2/100	ER2/20	RTU	Tonsil	Leica Microsystems	Buffalo Grove, IL
ER	1D5	ER1/20	1 : 50	Breast CA	Biocare Medical	Concord, CA
HER2	CB 11	ER1/20	RTU	Breast CA	Dako	Carpinteria, CA
Ki-67	MIB-1	ER1/20	1 : 80	Tonsil	Dako	Carpinteria, CA
P53	DO-7		1 : 40	S95-13083	Dako	Carpinteria, CA
PR	PgR 636	ER1/10	1 : 400	Endo/Myome	Dako	Carpinteria, CA

**Table 2:** Distribution of clinicopathologic data of breast cancer cases.

Age distribution	Mean (years)	Standard deviation (years)
	49.34	13.739
*Histological diagnoses*	Type	Frequency (percentage)
	Invasive carcinoma NST	166 (83.0)
	Ductal carcinoma in situ (DCIS)	9 (4.5)
	Metaplastic carcinoma	6 (3.0)
	Invasive lobular carcinoma	5 (2.5)
	Mucinous carcinoma	5 (2.5)
	Invasive papillary carcinoma	2 (1.0)
	Medullary carcinoma	2 (1.0)
	Others	5 (2.5)

*Histological grade*	Grade I	14 (9.4)
	Grade II	52 (34.9)
	Grade III	83 (55.7)

*Lymphovascular invasion*	Positive	32 (49.2)
	Negative	33 (50.8)

**Table 3:** Distribution of biomarker expressions in breast cancer patients.

Biomarker	Percentage expressed (%)
*bcl-2*	
Positive	37.9
Negative	62.1

ER	
Positive	29.0
Negative	71.0

PR	
Positive	10.9
Negative	89.1

HER2 *overexpression*	
Positive	20.7
Negative	79.3

*p*53	
Positive	36.7
Negative	63.3

*Ki-*67	
Low (mild)	62.1
Moderate	16.5
High (severe)	21.4

**Table 4:** Molecular classes of cases based on IHC staining.

	Frequency	Percent
Luminal A	32	19.8
Luminal B	16	9.9
HER2 overexpression	26	16.0
Triple negative	88	54.3

**Table 5:** Correlations of bcl-2 with molecular subtypes, apoptotic biomarkers, and clinicopathologic characteristics.

bcl-2				
	% Positive	Cramer's V	Pearson's Chi^2^	Sig. (*p*)
*Molecular subtypes*				
Luminal A	45.2	0.005	0.004	0.950
Luminal B	**75**	**0.204**	**6.629**	**0.015**
HER2 positive	53.8	0.082	1.063	0.303
Triple negative	**36**	**0.174**	**4.856**	**0.038**

*Apoptotic biomarkers*				
p53	44.4	0.124	2.593	0.107
Ki-67 mild (low) Moderate High (severe)	54.1 18.9 27	0.121	2.583	0.275

*Clinicopathologic characteristics*				
Histol. grade I II III	38.5 44.7 34.3	0.099	1.284	0.526

Lymphovascular invasion	59.3	0.148	1.187	0.276

**Table 6:** Association of p53 and Ki-67 with molecular subtypes and clinicopathologic characteristics.

p53				
	% Positive	Cramer's V	Pearson's Chi^2^	Sig. (*p*)
Molecular subtypes				
Luminal A	41.4	0.021	0.070	0.791
Luminal B	**86.7**	**0.320**	**15.709**	**<0.001**
HER2 positive	36.0	0.029	0.130	0.719
TNBC	**31.0**	**0.173**	**4.651**	**0.033**

Clinicopathological characteristics				
**Histol. grade** **I** **II** **III**	**16.7** **26.1** **45.7**	**0.230**	**6.781**	**0.034**
Lymphovascular invasion	42.9	0.199	2.094	0.148

Ki-67				

Molecular subtypes				
Luminal A	**M 24.5** **L 28.6**	**0.265**	**11.322**	**<0.01**
Luminal B	**H 3.1** **M 3.6** **L 34.3**	**0.429**	**29.628**	**<0.001**
HER2 positive	**H 13.3** **M 32.1** **L 11.4**	**0.200**	**6.467**	**<0.05**
TNBC	66.7	0.168	4.595	0.101

Clinicopathological characteristics				
Histol. grade				
I	L 76.9 M 15.4 H 7.7	0.111	3.274	0.51
II	L 66.7 M 14.6 H 18.8			
III	L 56.3 M 16.9 H 26.8			
Lymphovascular invasion	L 59.3 M 22.2 H 18.5	0.129	0.912	0.634
Association between p53 and Ki-67				

p53
Ki-67 low (mild)	**59.5**	**0.268**	**11.957**	**<0.01**
Moderate	**41.4**			
High (severe)	**27.7**			

## Data Availability

The Excel data used to support the findings of this study may be released upon application to the Committee on Human Research, Publication and Ethics of School of Medical Sciences/Komfo Anokye Teaching Hospital, at Block J, School of Medical Sciences, Kwame Nkrumah University of Science and Technology, Kumasi, Ghana.

## Abbreviations

IHC: Immunohistochemistry
TNBC: Triple negative breast cancer
ER: Estrogen receptor
PR: Progesterone receptor
HER2: Human epidermal growth receptor 2
NST: No special type
NPI: The Nottingham Prognostic Index.
